# HLA-G Polymorphisms Are Associated with Non-Segmental Vitiligo among Brazilians

**DOI:** 10.3390/biom9090463

**Published:** 2019-09-09

**Authors:** Luciana Veiga-Castelli, Maria Luiza de Oliveira, Alison Pereira, Guilherme Debortoli, Letícia Marcorin, Nádia Fracasso, Guilherme Silva, Andreia Souza, Juliana Massaro, Aguinaldo Luiz Simões, Audrey Sabbagh, Renata Cardili, Eduardo Donadi, Erick Castelli, Celso Mendes-Junior

**Affiliations:** 1 Departamento de Genética, Faculdade de Medicina de Ribeirão Preto, Universidade de São Paulo, Ribeirão Preto-SP 14049-900, Brazil; mluiza.oliveira21@gmail.com (M.L.d.O.); alisongonpereira@hotmail.com (A.P.); guilhermedebortoli@usp.br (G.D.); le.marcorin@gmail.com (L.M.); nadiadeaguiar@yahoo.com.br (N.F.); alsimoes@fmrp.usp.br (A.L.S.); 2 Departamento de Química, Laboratório de Pesquisas Forenses e Genômicas, Faculdade de Filosofia, Ciências e Letras de Ribeirão Preto, Universidade de São Paulo, Ribeirão Preto-SP 14040-901, Brazil; guivallesilva@hotmail.com (G.S.); ctmendes@ffclrp.usp.br (C.M.-J.); 3 Molecular Genetics and Bioinformatics Laboratory, Experimental Research Unit (UNIPEX), School of Medicine, São Paulo State University (UNESP), Botucatu, State of São Paulo 18618-687, Brazil; andreia18_souza@hotmail.com (A.S.); erick.castelli@gmail.com (E.C.); 4 Departamento de Clínica Médica, Faculdade de Medicina de Ribeirão Preto, Universidade de São Paulo, Ribeirão Preto-SP 14049-900, Brazil; jdblmassaro@gmail.com (J.M.); nahas-renata@uol.com.br (R.C.); eadonadi@fmrp.usp.br (E.D.); 5 UMR 216 MERIT IRD, Faculté de Pharmacie de Paris, Université Paris Descartes, Sorbonne Paris Cité, 75006 Paris, France; audrey.sabbagh@ird.fr

**Keywords:** HLA-G, vitiligo, polymorphism, DNA sequencing

## Abstract

(1) Background: Vitiligo is characterized by white patches on the skin caused by loss of melanocyte activity or the absence of these cells. The available treatments minimize the symptoms by retarding the process of skin depigmentation or re-pigmenting the affected regions. New studies are required for a better comprehension of the mechanisms that trigger the disease and for the development of more efficient treatments. Studies have suggested an autoimmune feature for vitiligo, based on the occurrence of other autoimmune diseases in vitiligo patients and their relatives, and on the involvement of genes related to the immune response. (2) Methods: We evaluated, by massive parallel sequencing, polymorphisms of the *HLA-G* gene in vitiligo patients and control samples, to verify if variants of this gene could influence the susceptibility to vitiligo. (3) Results: We detected an association with non-segmental vitiligo regarding the haplotype Distal-010101a/G*01:01:01:01/UTR-1, adjusting for population stratification by using ancestry-informative markers (AIMs). (4) Conclusions: It remains unclear whether the *HLA-G* variants associated with vitiligo were detected because of the high linkage disequilibrium (LD) with HLA-A*02, or if the *HLA-A* variants previously reported as associated with vitiligo were detected because of the high LD with HLA-G*01:01:01:01/UTR-1, or if both genes jointly contribute to vitiligo susceptibility.

## 1. Introduction

Vitiligo is characterized by skin depigmentation due to lack of melanocyte function or loss of melanocytes in the advanced stage of the disease, affecting people from various ethnic backgrounds. Its prevalence is around 1% in the United States and in Europe, but ranges from less than 0.1% to greater than 8% worldwide [1]. Vitiligo can be classified into two main types, segmental and non-segmental. Segmental vitiligo is less common, affecting 5%–16% of the cases; it has a unilateral distribution, it tends to occur at a younger age, with 87% of the cases diagnosed before 30 years, and it presents a better prognostic, possibly resulting from a somatic mosaicism effect. Non-segmental vitiligo, the most common form, has symmetrical and bilateral distribution and can occur at any age [2]. Although its cause has not been established yet, there is evidence of a major autoimmune component, including the occurrence of autoantibodies against melanin in the affected individuals [3]. This theory is partially based on the higher frequency of other autoimmune diseases within vitiligo patients and their family members [4] and also on previous studies indicating that vitiligo is one of the predisposing factors for autoimmune/autoinflammatory diseases, such as thyroid diseases, rheumatoid arthritis, psoriasis, later onset insulin-dependent diabetes mellitus, systemic erythematous lupus, among others [5]. Furthermore, genetic, neurological, metabolic and environmental factors have already been reported [6]. Due to this complex etiology, the available treatments to mitigate the effects or to delay the progress of the disease are poorly efficient with different outcomes for each patient.

Studies have shown that several genes associated with vitiligo are involved in the immunological function. For instance, HLA alleles have been associated with susceptibility to vitiligo in different populations [7,8], including admixed ones such as Brazilians [9]. Many studies revealed that the HLA-A*02:01 allele is involved with vitiligo, presenting multiple vitiligo melanocyte autoantigens [10,11,12]. However, a recent study has shown that the regulation of its expression is also involved in vitiligo predisposition. For instance, a specific haplotype in a region located ~20-kb downstream *HLA-A* acts as an enhancer and is involved with a larger *HLA-A* mRNA expression. Such haplotype is in strong linkage disequilibrium with the HLA-A*02:01:01:01 allele, which led the authors to conclude that combined qualitative (i.e., the presence of HLA-A*02:01 specificity) and quantitative (a higher *HLA-A* mRNA expression) effects are involved in vitiligo risk [13]. HLA class II genes are also involved in vitiligo risk. HLA-DRB1*07 was associated with vitiligo in Brazil [9], the Netherlands [7] and India [14]. Similarly to *HLA-A*, an enhancer located between *HLA-DRB1* and *HLA-DQA1* genes regulates their expression, leading to the conclusion that the expression level of HLA class II molecules is as or more important than antigen specificity for vitiligo risk [15].

However, these studies have only evaluated HLA loci involved in antigen presentation, *HLA-A*, *HLA-B*, *HLA-C*, *HLA-DRB1*, and *HLA-DQB1*, and little attention has been given to immunomodulatory genes such as *HLA-G*. Therefore, it is not clear whether *HLA-G* gene polymorphisms and ligands may influence the susceptibility to vitiligo.

HLA-G is an immunomodulatory molecule identified for the first time at the maternal-fetal interface, where a suitable immunologic tolerance is highly desirable for the fetus acceptance. However, the HLA-G molecule is also expressed in lower levels on the cell surface of cornea, pancreas and thymus, among others [16].

A well-recognized role of the HLA-G molecule is the inhibition of natural killer (NK) cells and cytotoxic T lymphocytes by its binding to leucocyte receptors on these cells’ surface, including ILT-2, ILT-4, CD160 (BY55) and KIR2DL4 (CD158d), inducing inhibitory thyrosine motifs [17]. Furthermore, HLA-G expression has been negatively correlated with vitiligo among Tunisians [18], a finding that reinforces the possible influence of HLA-G on vitiligo development.

The role of the *HLA* gene family in vitiligo has already been evaluated in several populations worldwide such as Han Chinese [19], Egyptian [20], Japanese [10], Moroccan [21] and Brazilian [9]. However, the possible association of *HLA-G* gene polymorphisms with vitiligo has been investigated so far only in the Korean population [22,23]. The first study focused on the rs1736936 SNP (position -1305) at the *HLA-G* promoter region and found a significant association of this polymorphism with non-segmental vitiligo [22]. By contrast, the authors did not find any association of *HLA-E* and *HLA-F* promoter polymorphisms with vitiligo in the same population. In the other study, the authors focused on a specific polymorphism of the *HLA-G* 3’untranslated region (3’UTR), specifically the 14-bp InDel and showed that it was significantly associated with the risk of developing non-segmental vitiligo in the Korean population, with a greater proportion of subjects with a homozygote INS/INS genotype in the vitiligo group compared to controls [23]. Although these *HLA-G* polymorphisms have been associated with non-segmental vitiligo, authors do not analyze the whole genetic diversity of the *HLA-G* gene.

Considering the low variability at the *HLA-G* coding region and the wide *HLA-G* expression variation in normal and pathological conditions, the magnitude of HLA-G expression depends on the gene regulatory regions, as well as on the secreted factors into the microenvironment [17]. Both regulatory segments of the *HLA-G* gene (5’URR and 3’UTR) have a high degree of genetic variability and could influence *HLA-G* expression in complex physiological processes, as previously described, including previous studies from our group [24,25,26,27,28,29,30,31]. Thus, additional *HLA-G* regulatory variation sites may influence the development of non-segmental vitiligo.

In this study we aimed to evaluate polymorphisms and haplotypes of the *HLA-G* gene by next generation sequencing (NGS), considering all regulatory segments and exons, in vitiligo patients and controls from a Brazilian population, to investigate whether variants of this gene could influence the susceptibility to vitiligo. Since Brazilians carry African, European and Native American ancestries, we have also evaluated the effects of ancestry background on vitiligo susceptibility.

## 2. Material and Methods

### 2.1. Population Sample

This study was approved in its ethical aspects by the “Comitê de Ética em Pesquisa” of our institution (Faculdade de Filosofia, Ciências e Letras de Ribeirão Preto, FFCLRP-USP) according to process CEP-FFCLRP CAAE n.25,696,413.7.0000.5407.

We studied 50 non-segmental vitiligo patients from the Ribeirão Preto region, located in the northern region of the State of São Paulo, southeastern Brazil, and followed up at the Dermatology Outpatient Clinic of the University Hospital of the Ribeirão Preto Medical School, University of Sao Paulo. These individuals, 33 women and 17 men, with ages ranging from 18 to 75 years, filled out a questionnaire, signed an informed consent form and donated a 10-mL blood sample, which was stored in a Vacutainer^®^ EDTA-containing tube. A total of 393 healthy individuals, 186 women and 207 men, with ages ranging from 18 to 80 years, were selected within the Ribeirão region for the control group and were submitted to the same procedures.

### 2.2. Extended HLA-G Analysis

DNA was extracted using the salting-out protocol. DNA quality was evaluated for integrity, purity and concentration by Agarose Gel Electrophoresis, NanoDrop spectrophotometry (Thermo Fisher Scientific Inc., Waltham, MA, USA) and Qubit™ dsDNA BR assay (Thermo Fisher Scientific Inc.) respectively.

The 5’URR (from −2638 to −1), coding sequence (CDS, only exons) and 3’UTR regions of *HLA-G*, in addition to other unrelated genomic regions of interest for the group, such as regions that include the SNP*for*ID 34-plex ancestry informative marker (AIM) set of SNPs [32], were selected for an in silico assay for probe design using the SureDesign tool (Agilent Technologies, Inc., Santa Clara, CA, USA), resulting in a set of probes capable of capturing a genomic region of 488,658 bp. However, this procedure did not capture the *HLA-G* segment between positions −546 and −369.

DNA Libraries were prepared with the HaloPlex Target Enrichment System (Agilent Technologies, Inc., Santa Clara, CA, USA) protocol, as described elsewhere [33]. Library quality was verified both by the 2100 Bioanalyzer (Agilent Technologies, Inc.), and the Qubit^®^ 2.0 Fluorometer (Thermo Fisher Scientific Inc., Waltham, MA, USA). The latter was also used for DNA quantification. A pool of DNA libraries, consisting of up to 96 samples (4 nmol/L) was diluted to 16 pM and inserted as input for sequencing using the MiSeq Reagent kit V3 (600 cycles), in the MiSeq Personal Sequencer (Illumina Inc., San Diego, CA, USA).

### 2.3. Bioinformatics Analysis

The *HLA-G* mapping, genotyping and haplotyping strategy used here has already been published elsewhere [30]. In brief, prior to mapping, all DNA segments (reads) produced by NGS were trimmed on both ends for primer and adapter sequences using the Cutadapt software. Mapping was performed by hla-mapper [34] version 2.2, function dna, database version 2.1, with the error threshold set to 0.03, minimum read size length set to 50 and the human hg38 used as a reference.

The Genome Analysis Toolkit (GATK, version 3.7) HaplotypeCaller was used for genotype calling in the GVCF (genomic variant call format) mode, applied for each sample separately [35]. Then, a VCF (variant call format) file was generated by the concatenation of all samples together with the GATK GenotypeGVCFs algorithm. Then, genotypes were processed by vcfx checkpl (www.castelli-lab.net/apps/vcfx) with the minimum genotype likelihood set to 99.9%, which guarantees that only high-quality genotypes are maintained for a further imputation step.

The physical association between alleles from different variation sites was assessed by inferring the physical phase among variants from the sequencing data using the GATK ReadBackedPhasing routine, coupled with a Bayesian probabilistic model implemented in the PHASE software [36], as described elsewhere [30]. The PHASE algorithm also imputed the missing alleles observed after the vcfx treatment. The scripts used to infer haplotypes are available online at https://github.com/erickcastelli/phase-readbackedphasing. It should be highlighted that all singletons have been removed before the haplotype inference step using the PHASE algorithm. Singletons were manually included later in the phased VCF file, when they met the following criteria: (a) there was no missing allele for any sample regarding this particular position; and (b) there was a straightforward relationship between the singleton and a neighboring heterozygous site, either visually detected using the BAM file of that sample or because it is the only heterozygous site detected on that particular sample.

The phased VCF file was converted into *HLA-G* CDS sequences using the hg38 reference sequence as a draft and replacing the correct nucleotide in each position, two sequences per sample, by using the application vcfx [function fasta] (www.castelli-lab.net/apps/vcfx). By using a local BLAST server with databases containing all known class I and II *HLA* CDS sequences described so far, downloaded from the IPD-IMGT/HLA database (https://www.ebi.ac.uk/ipd/imgt/hla/) version 3.31.0, the closest known *HLA-G* coding allele was defined for each haplotype.

### 2.4. Population Structure and Association Analysis

The SNP*for*ID 34-plex ancestry informative SNP panel [32] was employed to estimate the ancestral contribution on the samples of the present study, following the approach previously described [33]. Briefly, we mapped reads against the human reference genome (hg19) using BWA MEM, called genotypes using GATK UnifiedGenotyper (v.3.7), and treated genotypes with *vcfx checkpl*. We assessed the ancestry background of each sample using Structure 2.3.4 (https://web.stanford.edu/group/pritchardlab/structure.html), and the European, African and East Asian populations from the 1000 Genomes phase 3 as parental groups.

In order to account for population stratification in the association analysis, main ancestry components were included as covariates in a logistic regression model using PLINK v.1.9 (http://pngu.mgh.harvard.edu/purcell/plink/). At first, the association analyses were performed without taking the ancestry information into account, and, afterward, these statistics were recalculated after adjustment for ancestry, therefore avoiding spurious associations due to population stratification bias.

## 3. Results

**Figure 1:**
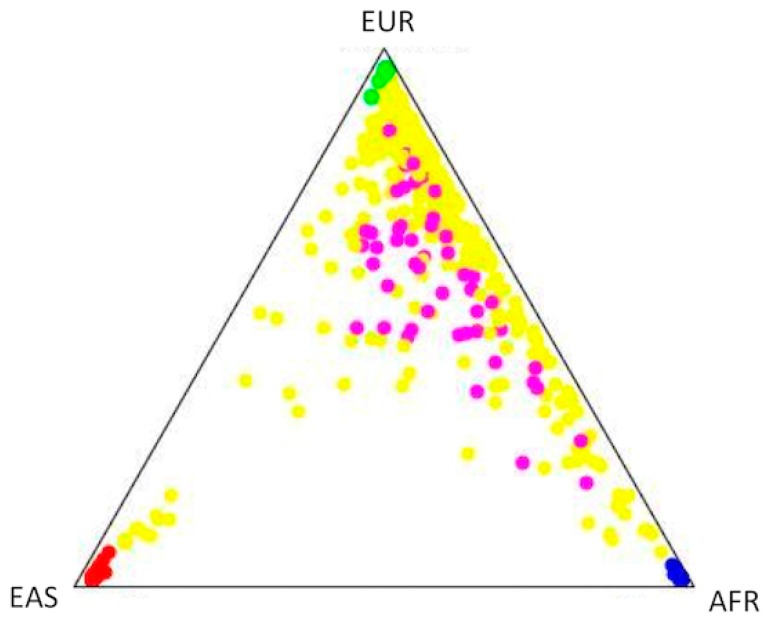
Genomic ancestry distribution in a Brazilian population sample composed of vitiligo patients and healthy controls. This triangular plot was generated by the Structure software, and the corners correspond to parental populations: Africans (AFR), East Asians (EAS), and Europeans (EUR). Yellow (healthy controls) and pink (vitiligo patients) dots correspond to Brazilian individuals, while blue, green, and red dots represent Africans, Europeans, and East Asians, respectively.

The ancestry background of the vitiligo group was 58.5% European, 29.7% African, and 11.7% Asian/Amerindian (Figure 1). In contrast, the control group’s ancestry background was 72.3% European, 19.9% African, and 7.8% Asian/Amerindian (Figure 1). This indicates a higher African and lower European ancestry among Brazilian vitiligo patients when compared to controls.

Considering all 443 samples and the HaloPlex panel designed for this study, we detected and studied 105 variable sites distributed along the *HLA-G* locus fitting Hardy–Weinberg expectations (*p* ≥ 0.01, Table S1). Comparing patients with controls (Table 1, upper panel), we detected allele rs9380142/G associated with non-segmental vitiligo (*p* = 0.01645, OR = 2.096 for the dominant model). rs9380142 is located at the 3’UTR, at position +3187, with allele Guanine composing the 3’UTR haplotype previously described as UTR-1 [26].

When we considered ancestry as a confounding variable (Table 1, lower panel), rs9380142/G remains associated with non-segmental vitiligo in both additive and dominant models. UTR-1, associated with rs9380142/G, is in absolute LD with G*01:01:01:01 in Brazilian, African, and European populations [30,37,38], and also with the distal HLA-G promoter Distal-G010101a [33]. Although not shown here, this sample presented the same LD pattern. UTR-1 and the promoter Distal-G010101a are also associated with vitiligo when we consider ancestry as a confounding variable (data not shown).

When the Bonferroni correction is applied, none of the associations described above remains significant. However, considering that all the variable sites detected at the *HLA-G* gene are included in a very small gene segment, and they all present a significant linkage disequilibrium among pairs of these polymorphisms [27,37], the Bonferroni correction for multiple testing was not taken into account to adjust the significance levels in this case-control comparison.

## 4. Discussion

It has been reported in the literature that a number of genes associated with non-segmental vitiligo might play a role in the immunological function. The role of *HLA-G* polymorphisms, whose molecule presents an immunomodulatory feature, in the susceptibility to vitiligo is unknown. Higher HLA-G expression has been described in other skin disorders, including melanoma [39], psoriasis [40], and pemphigus vulgaris [41]. However, HLA-G expression in biopsy specimens has been negatively correlated with vitiligo among Tunisians [18]. It has been shown that the transcriptional regulation of HLA genes plays a major role in the risk of developing autoimmune diseases, including vitiligo [13,15].

Here we evaluated *HLA-G* variability in vitiligo patients and controls surveyed in southeastern Brazil, adjusting for population stratification by using AIMs. We detected that the extended haplotype Distal-G010101a/G*01:01:01:01/UTR-1 is associated with vitiligo. This haplotype has been described as a high HLA-G producer [28,31,42]. The mechanism underlying this high expressing profile is probably related to the presence of variants that influence mRNA stability and degradations. For instance, G*01:01:01:01/UTR-1 is the only haplotype with allele +3187G that decreases the length of an AU-rich motif at the HLA-G mRNA 3’UTR segment, increasing mRNA stability [25]. This haplotype also presents Cytosine at position +3142 (rs1063320), negatively influencing the binding of many microRNAs, including miR-148a-3p and miR-152 [24]. Moreover, this haplotype is associated with a unique promoter sequence, named PROMO-G010101a [27], and a distal promoter sequence named Distal-010101a [33]. G*01:01:01:01/UTR-1 is usually the most frequent haplotype among Europeans (33%) and Asians (35%), but not among Africans (20%), based on the frequency of the associated allele +3187G (Table S1). Since our patient group presented a higher African background when compared to controls, this association became evident only after the ancestry adjustment (Table 1). In a recent study, a homozygous genotype involving this same variant (+3187G/G) was found associated with epithelial ovarian cancer, and the authors suggested that the genotype seems to influence the progression and outcome of that particular disease [43].

Autoimmunity is caused by a tolerance breakdown and HLA-G is a tolerogenic molecule. HLA-G expression on affected tissue cells should lessen autoimmune manifestations [17]. Thus, assuming that G*01:01:01:01/UTR-1 is a high HLA-G producer, the associations observed here are not consistent with our hypothesis that a lower HLA-G production would result in a stronger autoimmune response and, therefore, would be associated with vitiligo. However, it should be mentioned that G*01:01:01:01/UTR-1 was associated with a higher soluble HLA-G production [28] or with a high HLA-G production in specific tumor or embryonic stem cell lines [31,42], and thus it is not clear whether this haplotype would be also related to this increased production in different microenvironments such as the skin and melanocytes. For instance, different 3’UTR sequences present different microRNA binding profiles [44], and these sequences could influence HLA-G expression depending on the microRNA environment. In addition, the −725G allele (rs1233334) at the *HLA-G* promoter region, which is clearly associated with recurrent abortions [45], was surprisingly associated with a higher HLA-G mRNA expression in functional in vitro assays [46]. Moreover, it has been shown that a substantial proportion of gene expression heritability is trans to a given structural gene, and several trans variants act predominantly in a tissue-restricted manner and may regulate the transcription of many genes [47]. This highlights the fact that gene expression is regulated in a tissue-specific manner, according to a very specific cellular microenvironment. So, the current knowledge regarding the availability of transcription factors, miRNAs and other regulatory molecules on different tissues, does not rule out the possibility of tissue-specific regulation of HLA-G expression, with a possible down-regulation of the haplotype carrying UTR-1 in the skin or the melanocyte, an outcome that would make the present results consistent with our hypothesis.

Another hypothesis is that HLA-G expressed on the skin of vitiligo patients interacts with KIR2DL4 receptors from NK cells, and this interaction may induce the production of proinflammatory cytokines such as IFN-γ, TNF-α, IL-1α, IL-1β, IL-6, and IL-8 [48]. This could be more consistent in patients carrying G*01:01:01:01/UTR-1. It has already been reported that both TNF-α and IL-1α are increased in non-segmental vitiligo lesions [49]. Moreover, non-segmental vitiligo patients also presented increased levels of IL-6 [50].

It should be mentioned that there is a high LD between haplotype G*01:01:01:01/UTR-1 and alleles HLA-A*02:01 [12]. *HLA-G* and *HLA-A* are separated by 111 Kb, and a previous study demonstrated a single segregation block (high LD) from *HLA-G* up to 20 Kb towards *HLA-A* [37]. In particular, the *HLA-A* gene is thought to play a role in the susceptibility to vitiligo, especially the allele group A2, and mainly the A*02:01 alleles. Such a role has already been described in India [8], China [11], Japan [10], United States (European ancestry) and Europe [51], and Brazil [9]. Most of the studies conducted so far evaluated non-admixed populations, with the exception of the latter which evaluated Brazilian samples.

The Brazilian study [9] evaluated patients and controls also from southeast Brazil. Ramire and colleagues evaluated allele frequencies of classical class I genes (*HLA-A*, *HLA-B*, and *HLA-C*) and class II genes (*HLA-DRB1* and *HLA-DQB1*). However, this previous study did not control for population stratification. Nevertheless, it also pointed to an association of HLA-A2 alleles with vitiligo. As previously addressed, this vitiligo association may be reflecting combined qualitative (i.e., the presence of HLA-A*0201 specificity) and quantitative (a higher HLA-A mRNA expression) effects [13].

## 5. Conclusions

Besides uncovering *HLA-G* as a possible causal gene involved in vitiligo pathogenesis, the present results may either reinforce the importance of HLA-A*02 alleles in the vitiligo pathogenesis, even in admixed populations, or refute the previous conclusion that HLA-A*02:01 is involved in this disease [8,9,10,11,51].

On the one hand, A*02:01 molecules can present many antigens derived from melanocyte proteins, triggering immune responses against these antigens [52,53,54]. On the other hand, allele G*01:01:01:01/UTR-1 encodes a well-recognized immune-modulatory molecule and has been associated with differential expression profiles.

To sum up, it becomes unclear whether (a) the association of *HLA-G* variants with vitiligo here reported was detected because of the high LD with HLA-A*02, configuring a hitchhiking effect; (b) the associations of *HLA-A* variants with vitiligo reported elsewhere [8,9,10,11,51] were detected because of the high LD with HLA-G*01:01:01:01/UTR-1, configuring an inverse hitchhiking effect; or (c) both genes jointly contribute to vitiligo susceptibility, since G*01:01:01:01/UTR-1 and A*02:01 are among the most frequent alleles worldwide.

## Figures and Tables

**Table 1:** Sites associated with vitiligo detected in a Brazilian sample.

Variant	HLA-G Position	Associated Allele	Model	OR	L95	U95	P	Known HLA-G Alleles or Haplotypes
**Before ancestry adjustment**								
rs9380142	3187	G	Dominant	2.096	1.145	3.838	0.0164	Distal-010101a, Proximal-010101a, G*01:01:01:01, UTR-1 *
**After ancestry adjustment**								
rs9380142	3187	G	Additive	1.590	1.020	2.479	0.0404	Distal-010101a, Proximal-010101a, G*01:01:01:01, UTR-1 *
rs9380142	3187	G	Dominant	2.270	1.222	4.217	0.0095	Distal-010101a, Proximal-010101a, G*01:01:01:01, UTR-1 *

* according to Castelli et al., 2010; Castelli et al., 2017; Oliveira et al., 2018.

## Supplementary Materials

The following are available online at https://www.mdpi.com/2218-273X/9/9/463/s1, Table S1: HLA-G variants detected in a Brazilian sample of vitiligo patients and non-vitiligo controls, and their frequencies in three population groups from the 1000 Genomes dataset.
